# Susceptibility of Different Egyptian Populations of *Tuta absoluta* (Meyrick) to Selected Insecticides and Associated Detoxification Enzyme Activities

**DOI:** 10.3390/insects17050493

**Published:** 2026-05-12

**Authors:** Ahmed M. M. Ahmed, Lenni Ramirez-Flores, Mohammed A. A. Saad, Ahmed A. Alsherbiny, Hosam A. Ezz El-Din, Reda E. Korat, Nihal M. M. Khalil Bagy, Verónica Andrade-Yucailla, Marcos Barros-Rodríguez

**Affiliations:** 1 Plant Protection Department, Faculty of Agriculture, Assiut University, Assiut 71526, Egypt; 2 Centro de Investigaciones Agropecuarias, Facultad de Ciencias Agrarias, Universidad Estatal Península de Santa Elena, La Libertad 240204, Ecuador; 3 Plant Protection Department, Faculty of Agriculture, Al-Azhar University, Assiut 71524, Egypt; 4 Piercing and Sucking Insects Department, Plant Protection Research Institute, Agriculture Research Center, Giza 12112, Egypt; 5 Department of Animal Nutrition and Rumen Biotechnology, Ruminant Feedlot Ranch–PROCESA, Street Playita–Estero Hondo, La Maná 050202, Ecuador

**Keywords:** *Tuta absoluta*, insecticide resistance, resistance ratio, cytochrome P450, glutathione S–transferase

## Abstract

The tomato borer, *Tuta absoluta* (Meyrick) (Lepidoptera: Gelechiidae), is a highly destructive invasive pest of tomato, and its ability to develop resistance to insecticides is well known. The susceptibility of three field populations from Egypt (Luxor, Assuit, and Giza) to the insecticides chlorantraniliprole, chlorfenapyr, indoxacarb, emamectin benzoate, and spinetoram was evaluated by leaf-dip bioassays. The second-instar larvae were exposed, and mortality was noted 24, 48, and 72 h later. Probit analysis was used to calculate lethal concentrations (LC_50_ and LC_90_) and resistance ratios (RR_50_). Furthermore, key detoxification enzymes (α-esterases, cytochrome P450 monooxygenases, and glutathione S-transferases) were also measured to identify possible metabolic resistance mechanisms. Considerable inter-population differences were observed. Detoxification enzymes (α-esterases, cytochrome P450 monooxygenases, and glutathione S-transferases) were measured. The Luxor population showed the highest LC_50_ values and resistance ratios, especially to chlorfenapyr and indoxacarb, suggesting low-to-moderate resistance, while the Giza population was the most susceptible. Emamectin benzoate and spinetoram provided excellent control of all laboratory and field strains.

## Simple Summary

*Tuta absoluta* (Meyrick) is a major tomato pest that has spread worldwide, with multiple reports of insecticide resistance. The toxicity of five insecticides, namely chlorantraniliprole, chlorfenapyr, indoxacarb, emamectin benzoate and spinetoram, against three Egyptian field populations (Luxor, Assiut, and Giza) were evaluated through a leaf-dip bioassay on second-instar larvae. Probit analysis was used to calculate LC_50_, LC_90_ and relative resistance values based on mortality at 24, 48, and 72 h. Detoxifying enzymes (α-esterases, cytochrome P450 monooxygenases, glutathione S-transferases) were also observed. The highest LC_50_ value and resistance ratios were found in the Luxor strain, while Giza demonstrated was considered the most susceptible strain. Emamectin benzoate and spinetoram gave excellent control of all the laboratory and field strains tested. High metabolic-based resistance was inferred by increased P450 and GST activity in Luxor.

## 1. Introduction

Tomato (*Solanum lycopersicum* L.) is one of the most widely grown and consumed vegetables worldwide, as a source of vitamins and minerals, and it is considered a functional food [1]. A total of 95% of tomato fruit is water, and the remaining 5% consists of carbohydrates, fat, and protein [2,3,4]. They are rich in antioxidants as well as bioactive compounds, such as lycopene, that may protect against carcinogenic toxins. Tomatoes are eaten fresh, cooked, and processed. *Tuta absoluta* (Meyrick) (Lepidoptera: Gelechiidae), known as the South American tomato pinworm or tomato leaf miner, is an important pest of solanaceous crops, particularly tomato. It was initially found in Eastern Spain in 2006 but shortly thereafter spread quickly worldwide throughout the Mediterranean basin, Africa and Asia [5]. *T. absoluta* has been reported as a serious insect pest since 2009 in tomato fields in Egypt [6]. It was initially introduced into potato and tomato fields in the Marsa Matrouh governorate, near the Libyan border. Until 2010, it spread up to the Giza governorate, and then all the governors proceeding until it reached the Northern Sudanese border by June 2011 [7]. The larvae of *T. absoluta* attack the leaves, flowers, stems and fruits of tomato plants at all growth stages [8]. In certain parts of Egypt, this pest can result in crop losses of up to 100%. Both open-field and greenhouse tomato production are severely affected [9,10]. The major strategy for controlling *T. absoluta* populations is still chemical control [11]. However, in addition to eliminating natural enemies and increasing occupational hazards, overuse of insecticides, up to 36 sprays per cultivation period and occasionally two sprays per day, has resulted in the emergence of resistant populations [12,13]. According to earlier reports, *T. absoluta* is resistant to several pesticide families, including organophosphates (OPs), pyrethroids, abamectin, cartap, spinosad, and diamides [14]. More recent research has shown resistance to the oxadiazine indoxacarb, the diamide chlorantraniliprole, and spinosyns such spinosad [15]. Insecticide resistance in *T. absoluta* populations has been found to be largely caused by metabolic resistance brought on by the amplification of specific detoxification enzymes, such as esterases, glutathione S-transferases (GSTs), cytochrome P450 monooxygenases, and hydrolases [16]. The current study intends to assess the toxicity of five specific commercial insecticides against various *T. absoluta* populations collected from three locations in Egypt and to investigate the related biochemical mechanisms of resistance, such as the activities of cytochrome P450 monooxygenases, esterases, and GSTs. This study sheds light on resistance management techniques for tomato production that are sustainable.

## 2. Materials and Methods

### 2.1. Insecticides

Five commercial insecticides representing different chemical groups and modes of action were evaluated based on their widespread use in Egypt and relevance in resistance management. The pesticide obtained from Central Agricultural Pesticide Laboratory (CAPL) in Dokki, Giza, Egypt: Chlorantraniliprole (diamide, IRAC 28), Chlorfenapyr (pyrrole, IRAC 13), Indoxacarb (oxadiazine, IRAC 22), Emamectin benzoate (avermectin, IRAC 6), Spinetoram (spinosyn, IRAC 5). These insecticides were selected because they represent the most commonly used chemical groups in Egyptian tomato production and are recommended in resistance management programs due to their different modes of action, which reduce cross-resistance risk.

### 2.2. Rearing of T. absoluta

Larvae were collected from infested tomato fields in Luxor, Assiut, and Giza and were identified morphologically. Colonies were reared for one generation (F1) under laboratory conditions (25 ± 2 °C, 60–65% RH, 16:8 L:D) on Ellisa variety of tomato plants *Lycopersicon esculentum* (Mill.), (30–40 days old), and leaves were replaced every 2–3 days.

### 2.3. Bioassay

Bioassays of five commonly used insecticides, chlorantraniliprole, chlorfenapyr, indoxacarb, emamectin benzoate, and spinetoram, were performed on the second-instar larvae from laboratory colonies. Larvae were acclimatized for 24 h prior to testing. Each treatment included three replicates, with ten larvae per replicate.

The bioassay of insecticides on *T. absoluta* was carried out by leaf-dip bioassay method according to IRAC test method No. 022 [17]. The control leaves were dipped in the distilled water without insecticide, whereas other leaves were dipped in different insecticide solutions for 3 s with agitation, followed by drying at room temperature. The non-ionic surfactant Tween 80^®^ at a concentration of 500 ppm was used as the wetting agent in all treatments, including controls. The bottom of each Petri plate was covered with a wetted cotton layer and filter paper, and each Petri plate was labeled according to the solution. After shade drying, the individual leaves were placed in the Petri plates according to concentration. Finally, ten second-instar larvae per replication were placed on the leaves. Second-instar larvae were selected according to IRAC guidelines due to their uniform susceptibility and suitability for toxicity evaluation. After exposure of larvae to different insecticide doses, bioassay trays were stored at a temperature of 25 ± 2 °C, 60–65% RH and 16:8 light/dark photoperiod regimes. Mortality was recorded at 24, 48, and 72 h using a soft brush. Individuals showing complete immobility were considered died. Data was corrected for control mortality [18]. The LC_50_ and LC_90_ values were calculated by probit analysis using IBM SPSS statistics 20 software. LCP lines were drawn by using the program Sigma plot 8.02 system software. Resistance ratios (RR50) were calculated by dividing the (LC_50_) value of each field population by the (LC_50_) value of the most susceptible *T. absoluta* population for each insecticide [19].

### 2.4. Biochemical Analysis: Preparation of Homogenate Larvae

Fourth-instar larvae were used for enzyme assays due to their higher biomass and sufficient protein content required for biochemical analysis. Samples of ten fourth-instar *T. absoluta* larvae were collected from the reared colonies in the enclosed cages and homogenized in 1 mL Sodium Phosphate buffer (0.1 M pH7) using a Teflon glass homogenizer and centrifuged Eppendorf^®^ Centrifuge 5425 R (Eppendorf AG, Hamburg, Germany) at 10,000 rpm for 15 min at 4 °C (five replicates of each sample). The supernatant was used as a source of enzyme.

#### 2.4.1. Total Content of Proteins

Total protein content was determined based on the Biuret test [20], using a Kit purchased from DP International Laboratory (Giza, Egypt), a local manufacturer. A mixture of 1.0 mL of the total protein reagent (0.2 N sodium hydroxide, 18 mM/L sodium potassium tartrate, 12 mM/L potassium iodide and 6 mM/L cupric sulfate), 20 μL of each sample and 20 μL of deionized water was then incubated for 5 min at 25 °C. The absorbance values of the sample (A) and standard protein (Ά) were measured by Unico Spectrophotometry (Model No. UV2802) (Dayton, NJ, USA) against the blank at a wavelength of 550 nm. Total protein in the sample was calculated as (mg/g body weight of larvae) using Equation (1):

Total protein (gm/dL) = (A/Ά) × 6 (standard Conc.)(1)

#### 2.4.2. Determination of α-Esterases Activities

The activities of α-esterases were determined according to the method described by Van Asperen [21] using α–naphthyl acetate as substrates. Alpha–naphthyl acetate substrates were hydrolyzed by esterase enzymes to form α–naphthol. The produced alpha–naphthol was converted by adding diazoblue B sodium lauryl sulphate solution [prepared by mixing 2 parts of 1%Diazoblue and 5 parts of 5% sodium lauryl sulphate (SDS)] to form strong blue colors which may be spectrophotometrically measured at 600 nm wavelengths. Sodium lauryl sulphate strongly enhanced the color produced. Esterase activity was determined using the extinction coefficient of [22] based on total volume. Esterase activity was calculated by dividing optical density (OD)/min by the extinction coefficient to get nMole/min. Specific esterase activity was obtained by dividing esterase activity by total protein (mg/mL) in each sample to get nMole/min/mg protein.

#### 2.4.3. Determination of Cytochrome P450 Monooxygenases Activity Towards p-Nitro Anisole (p-NA)

Assay of O–demethylation of p-nitroanisole (PNOD) activity by CYP450s was determined according to the method of [23]. Reactions were carried out in 96-well micro-plates by monitoring p-nitrophenol formation in a final volume of 200 μL at 405 nm using p–nitroanisole (p-NA) as a substrate at 30 s intervals for 15 min at 30 °C. Each reaction mixture contained 100 mM potassium phosphate buffer, pH: 7.4, 0.5 mM NADPH, 1 mM p–NA and 90 μg proteins in a final volume of 200 μL. The molar extinction coefficient for p-nitrophenol at 405 nm was determined by preparing standard curves and was used to calculate CYP450–PNOD activities as pmole/min/mg protein.

#### 2.4.4. Determination of GST Enzyme Activity Towards 1-Chloro-2,4–Dinitrobenzene (CDNB)

According to Habig et al. [24], the assay was conducted by incubating 50 mM of CDNB (1-chloro-2,4-dinitrobenzen) as a substrate with 50 mM GSH (reduced glutathione) and 50 μL of sample enzyme in 0.1 M of phosphate buffer (pH7) for 5 min at 27 °C (five replicates for each sample). The activity was measured at 340 nm, and Specific GST activity was calculated as nMole/min/mg protein using Equation (2).

Specific activity = {[(∆A340)/min] × V × 1000}/(ɛmM × Venz × Pc)(2)

where

∆A340/min = Change in absorbance per minute, V = The reaction volume (2.5 mL), Pc= Protein concentration (mg/mL), Venz = Volume of enzyme (0.05 mL), and ɛmM = Extinction coefficient for CDNB at 340 nm (9.6 mM/mL).

### 2.5. Statistical Analysis

Data was analyzed using one-way analysis of variance (ANOVA). Assumptions of normality and homogeneity of variance were tested using Shapiro–Wilk and Levene’s tests, respectively. When ANOVA indicated significant differences, means were separated using Duncan’s Multiple Range Test (DMRT) at *p* ≤ 0.05. These analyses were performed using CoStat Version 6.303 [25]. LC_50_ and LC_90_ values, along with their 95% fiducial limits (FL) and slope values, which were estimated using probit analysis in SPSS software (Version 26.0). Significant differences were determined based on non-overlapping 95% confidence intervals at *p* < 0.05.

## 3. Results

### 3.1. Toxicity and Resistance Ratio of Chlorantraniliprole on Different Populations of T. absoluta Under Laboratory Conditions

The results obtained from (Table 1) led to the Luxor population recording the highest values of LC_50_ and LC_90_ after 24, 48, and 72 h of exposure to chlorantraniliprole; it recorded 0.79, 5.03, 0.51, 2.54, 0.35 and 1.68 μg/mL for LC_50_ and LC_90_ after 24, 48, and 72 h, respectively. Therefore, it recorded the highest resistance ratio RR_50_ and RR_90_ after 24, 48 and 72 h of exposure at 2.5-, 2.0-, 2.2-, 2.1-, 1.8- and 2.1-fold, respectively. The Assuit population followed, which recorded 0.28, 2.31, 0.19, 1.27, 0.18, and 0.87 μg/mL for LC_50_ and LC_90_ after 24, 48 and 72 h, respectively. Also, it recorded relative resistance RR_50_ at 1.2-, 1.1- and 1.1-fold after 24, 48 and 72 h, respectively, and RR_90_ at 2.0-, 1.7- and 2.1-fold after 24, 48 and 72 h of exposure, respectively, compared to the Giza population, which recorded the lowest values of LC_50_ and LC_90_.

### 3.2. Toxicity and Resistance Ratio of Chlorfenapyr on Different Populations of T. absoluta Under Laboratory Conditions

Data obtained from (Table 2) led to the Luxor population recording the highest values of LC_50_ and LC_90_ after 24, 48, 72 h of exposure to chlorfenapyr; it recorded 126.76, 268.12, 301.48, 94.19, and 263.65 μg/mL, respectively, followed by the Assuit population, which recorded 98.65, 196.77, 85.86, 163.994, 70.95 and 134.086 μg/mL, respectively. Compared to the Giza population, Giza recorded the lowest values of 47.156 114.26, 46.67, 140.87, 39.34 and 99.29 μg/mL of LC_50_ and LC_90_ after 24, 48 and 72 h of exposure to chlorfenapyr, respectively. A clearly high relative resistance was recorded by the Luxor population at LC_50_ of 3.1, 3.02 and 3.4 after 24, 48 and 72 h, respectively; this relative resistance decreased at LC_90_ of 1.8, 1.4 and 1.8 after 24, 48 and 72 h, respectively, compared to the Giza population. Also, the Assuit population recorded a relative resistance at LC_50_ of 2.1-, 1.8- and 1.8-fold after 24, 48, 72 h respectively. It decreased at LC_90_ by 1.7-, 1.2- and 1.4-fold after 24, 48 and 72 h, respectively, compared to the Giza population.

### 3.3. Toxicity and Resistance Ratio of Indoxacarb on Different Populations of T. absoluta Under Laboratory Conditions

As shown in (Table 3), it was revealed that the toxicity of indoxcarb on Luxor and Assuit populations was affected by levels of relative resistance at RR_50_ and RR_90_ compared to the Giza population. LC_50_ and LC_90_ of the Luxor population recorded 139.50, 187.77, 116.08, 175.50, 107.03, 107.03 and 160.47 μg/mL after 24, 48, and 72 h of exposure, respectively. Conversely, the Assuit population recorded 95.53, 227.99, 83.34, 200.26, 68.64 and 151.58 μg/mL for LC_50_ and LC_90_ after 24, 48, and 72 h of exposure, respectively. However, the Giza population recorded 43.36, 104.01, 38.07, 77.77, 33.66 and 64.59 μg/mL for LC_50_ and LC_90_ after 24, 48, and 72 h of exposure, respectively. Therefore, the Luxor population recorded the highest relative resistance RR_50_ of 3.2-, 3.0- and 3.2-fold and RR_90_ of 1.8-, 1.3- and 2.5-fold after 24, 48, and 72 h, respectively. Following this, the Assuit population recorded RR_50_ 2.2, 2.2 and 2.0 and RR_90_ 2.2-, 2.6- and 2.3-fold after 24, 48, and 72 h, respectively.

### 3.4. Toxicity and Resistance Ratio of Emamectin Benzoate on Different Populations of T. absoluta Under Laboratory Conditions

Data shown in (Table 4) shows that LC_50_ and LC_90_ for the Luxor population were 0.56, 2.73, 0.37, 1.83, 0.26 and 1.24 μg/mL after 24, 48, and 72 h of exposure, respectively. Following this, the Assuit population recorded 0.33, 2.51, 0.22, 1.69, 0.18 and 0.77 μg/mL for LC_50_ and LC_90_ after 24, 48, and 72 h of exposure, respectively. However, the Giza population recorded the lowest values of 0.27, 1.43, 0.21, 1.0, 0.18, 0.75 μg/mL for LC_50_ and LC_90_ after 24, 48, and 72 h of exposure to emamectin benzoate, respectively. Therefore, the Luxor population recorded relative resistance RR_50_ of 2.1-, 1.8- and 1.4-fold and RR_90_ of 1.9-, 1.8-, and 1.7-fold after 24, 48, 72 h, respectively. The Assuit population followed, recording RR_50_ of 1.2-, 1.1-, and 1-fold and RR_90_ of 1.7-, 1.7- and 1.03-fold after 24, 48, and 72 h respectively, compared to the Giza population.

### 3.5. Toxicity and Resistance Ratio of Spinetoram on Different Populations of T. absoluta Under Laboratory Conditions

Data illustrated in (Table 5) shows that the Luxor population recorded LC_50_ and LC_90_ of 1.87, 11.48, 1.33, 6.75, 0.94 and 3.2 μg/mL after 24, 48 and 72 h of exposure, respectively. Significant differences among populations were observed only for chlorfenapyr and indoxacarb, where non-overlapping 95% confidence limits indicated statistical significance. However, overlapping confidence limits for chlorantraniliprole, emamectin benzoate, and spinetoram indicate no significant differences among Luxor, Assiut, and Giza populations.

### 3.6. Enzyme Activities in Different Populations of Tuta absoluta

**Figure 1:**
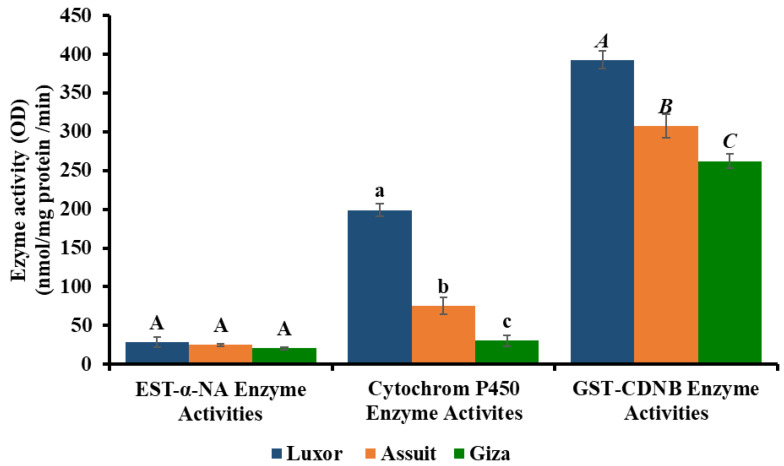
Relative increase in α-esterases, Cytochrome P450 (PCMAN–demethylase monooxygenase), and GST (CDNB) enzyme activities in different populations of *Tuta absoluta*. Different letters indicate significant differences among populations (DMRT, *p* ≤ 0.05).

Results displayed in (Table 6) show that the Luxor population recorded the highest values of the tested enzymes activities (esterases, CytochromP450 and Glutathion S-transferse), compared to the Giza population which recorded the lowest values. One-way ANOVA revealed significant differences in cytochrome P450 and GST activities among populations, while α-esterase activity showed no significant differences (Table 7). Date in (Table 6) and (Figure 1) recorded relative increases of 1.4-, 6.5- and 1.5-fold in the activity of the esterases, CytochromP450 and GST enzymes, respectively, followed by the Assuit population which recorded increases of 1.2-, 2.5- and 1.2-fold, respectively; compared to the Giza population, Giza recorded the lowest values. It could be concluded that the Giza population recorded the lowest values of LC_50_ and LC_90_, as well as the lowest values of all enzyme activities. Conversely, the Luxor population was found to be resistant to the five tested insecticides and demonstrated an increase in the activity of the studied enzymes EST-α-NA, Cytochrom P450, GST (Glutathion S-transferse) enzyme, suggesting a possible association between detoxification enzyme activities and insecticide susceptibility and the studied detoxifying enzymes. Meanwhile, the Assuit population was resistant to indoxacarb and chlorfenapyr, with an increase in the activity of Cytochromp450 and GST enzymes. There was no significant difference in EST enzyme activity among the Assuit and Giza populations, suggesting that the EST enzyme has no role in the indoxacarb and chlorfenapyr resistance mechanisms, while Cytochrom P450 and GST play a major role in the resistance mechanism.

## 4. Discussion

The previous results indicated that the Giza population recorded the lowest values of LC_50_ and LC_90_ for the tested insecticides (chlorantraniliprole, chlorfenapyr, indoxacarb, emamectin benzoate and spinetoram). The Luxor and Assiut populations exhibited significantly low to moderate resistance only to chlorfenapyr and indoxacarb, as indicated by non-overlapping 95% confidence limits. In contrast, overlapping confidence limits for chlorantraniliprole, emamectin benzoate, and spinetoram suggest no significant differences compared to the Giza population. The higher resistance observed in the Luxor population compared to Giza may be attributed to differences in insecticide exposure history and local agricultural practices. Intensive and repeated use of insecticides in certain regions can lead to the selection of resistant individuals. In contrast, lower selection pressure in Giza may explain the higher susceptibility observed. Additionally, the elevated activity of detoxification enzymes such as cytochrome P450 monooxygenases and glutathione S-transferases in the Luxor population supports the role of metabolic resistance mechanisms.

Similar findings have been reported previously. For example, one study evaluated nine Cypriot populations under laboratory conditions from 2016 to 2018 and found that chlorantraniliprole and indoxacarb no longer provided effective control. Resistance ratios exceeded 28-fold for chlorantraniliprole and ranged between 3- and 23-fold for indoxacarb [26]. Furthermore, mortality achieved by those two insecticides was 20.6% to 72% for chlorantraniliprole and 27.5% to 78% for indoxacarb. However, the insecticides emamectin benzoate and spinosad are very effective, since mortality to both of them ranged between 99.5% and 100%. Reports from Greece further confirm emerging resistance to diamide insecticides in *T. absoluta*. Resistance ratios reached up to 14-fold for chlorantraniliprole and 11-fold for flubendiamide, suggesting that although diamide resistance is still relatively low, it increases over time and requires continuous monitoring [27]. Low levels of resistance to chlorfenapyr have also been reported but generally remain limited, likely due to restricted use of this compound [28]. In Brazil, resistance to abamectin and cartap has been documented across all tested populations, with resistance ratios of 5.2–9.4-fold and 2.2–21.9-fold, respectively [29]. The resistance ratio for abamectin and cartap ranged from 5.2- to 9.4-fold and from 2.2- to 21.9-fold, respectively. One study mentioned that insecticide emamectin benzoate is an extremely potent pest control tool; therefore, it is extensively used in current IPM schemes for *T. absoluta*. However, the investigation of a total of 35 *T. absoluta* populations collected between 2012 and 2016 from Greece, Italy, Spain, Palestine and the UK indicated six cases of low to moderate resistance to emamectin benzoate, with resistance ratios greater than 15-fold. Reference [30] reported that spinosad and spinetoram resistance reached significant levels and is now common in the Antalya population. Reference [31] studied the susceptibility to spinetoram for eight representative populations of *T. absoluta* during 2010 and 2011 in four regions of Brazil and found that the LC_50_ values varied from 0.047 to 0.308 mg/L. The resistance levels ranged from 1.0- to 6.5-fold (RR_50_) and 1.0- to 12.1-fold (RR_90_) [32].

Compatible results were obtained by [19], who found that CYP450–PNOD activities showed 2.55- and 1.95-fold increases compared to susceptible populations in Adana and Antalya field populations, respectively. Furthermore, GST–CDNB activities showed a statistically significant (*p* < 0.05) 1.3-fold increase only in the Adana population. Although EST-alpha-NA activities showed a 3.41-fold increase only in the Ankara field population, this field population did not display significant resistance to abamectin, concluding that cytochrome P450 monooxygenase enzymes seemed to have a major role in abamectin resistance development in field populations of *T. absoluta* from Turkey. In addition, GSTs possibly have a supportive role such as reducing oxidative stress that developed during metabolism of abamectin in resistant field populations of *T. absoluta*. Also, ref. [28] conducted a survey of the susceptibility to indoxacarb, metaflumizone, chlorfenapyr, cartap, and abamectin and aimed to determine the resistance status of *T. absoluta* populations. Also, the major enzyme systems associated with metabolic resistance were assessed to infer variability. The LC_50_ values varied among the populations for abamectin (0.54 to 3.38 mg a.i./L), cartap (93.1 to 589.8 mg a.i./L), chlorfenapyr (0.62 to 2.83 mg a.i./L), indoxacarb (0.86 to 2.89 mg a.i./L), and metaflumizone (0.35 to 7.44 mg a.i./L). Resistance ratios varied among populations, being 6.2, 6.4, 4.6, 3.3, and 21.2 times higher for abamectin, cartap, chlorfenapyr, indoxacarb, and metaflumizone, respectively. Only the cartap confidence limits of the LC_80_ bracketed the recommended label concentration for three populations (An apolis, Guaraciaba do Norte, and Tiangua), suggesting control failures. No cross-resistance was observed among indoxacarb and metaflumizone, and natural variation may explain the variability of response to this latter insecticide. The activity of enzymes frequently associated with metabolism of insecticides significantly differed among populations, and glutathione S-transferases and cytochrome P450-dependent monooxygenases were variable among the populations of *T. absoluta*, while alpha- and beta-esterases were very homogeneous. Another compatible result was obtained by [33], who studied the resistance of two (Aydın and Urla) populations of *T. absoluta* against five commonly used insecticides (indoxacarb, spinosad, azadirachtin, chlorantraniliprole and metaflumizone). Further, the activity of insecticides detoxifying enzymes [gluthation–S–transferase (GST) and esterase (EST)] was also evaluated to confirm resistance. The Aydın population of *T. absoluta* had higher resistant values by 8-fold, 3.79-fold, 6.4-fold and 1.84-fold for indoxacarb, metaflumizone, spinosad and chlorantraniliprole, respectively, against all insecticides except azadirachtin, compared to the Urla population. It was determined that in comparison with the *T. absoluta* population from Aydın, the Urla population can be more susceptible to other insecticides except azadirachtin. GST enzyme activity was 1.5-fold higher in Aydın than the Urla population; however, the EST enzyme showed similar activity in both populations. The results of the study imply that *T. absoluta* populations from Aydın (Turkey) can be resistant against indoxacarb, metaflumizone, spinosad and chlorantraniliprole. Increased GST enzyme activity in resistant populations confirms this resistance development. Insecticides of plant origin like azadirachtin, for which the lowest insecticide resistance was recorded, may be applied in combination with other methods to effectively control *T. absoluta* [33]. The use of different larval instars for bioassays and biochemical analysis may influence the interpretation of resistance mechanisms. The -second-instar larvae were selected for bioassays due to their higher susceptibility to insecticides, while fourth-instar larvae were used for enzyme assays due to their larger body size and higher metabolic activity. The use of different larval instars among bioassays and biochemical assays may influence enzyme expression levels; however, elevated detoxification enzyme activity in the Luxor population still reflects true metabolic resistance.

## 5. Conclusions

The results showed marked variation in susceptibility among populations of *Tuta absoluta*, with some exhibiting high levels of resistance. Elevated activity of detoxifying enzymes, including cytochrome P450 and GST, suggests that metabolic resistance is one of the major mechanisms. These results highlight the need to rotate insecticides, monitor resistance, and implement integrated pest management (IPM) strategies to preserve effective control and prevent additional resistance development.

## Figures and Tables

**Table 1:** Toxicity and resistance ratio of chlorantraniliprole on different populations of *Tuta absoluta* 2nd instar larvae under laboratory conditions after 24, 48 and 72 h of exposure.

Population	LC_50_ (μg/mL) (95% CL)	LC_90_ (μg/mL) (95% CL)	Slope ± SE	RR_50_	RR_90_
After 24 h					
Luxor	0.79 (0.376–1.348)	5.03 (2.569–26.673)	1.596 ± 0.404	2.5	2
Assuit	0.28 (0.083–0.509)	2.31 (1.112–20.881)	1.403 ± 0.413	1.2	2
Giza	0.25 (0.102–0.393)	1.12 (0.655–4.411)	1.943 ± 0.533	1	
After 48 h					
Luxor	0.51 (0.224–0.835)	2.54 (1.441–10.007)	1.843 ± 0.482	2.2	2.1
Assuit	0.19 (0.346–0.43)	1.27 (0.675–8.064)	1.570 ± 0.483	1.1	1.7
Giza	0.181 (0.055–0.29)	0.72 (0.439–3.084)	2.127 ± 0.665	1	
After 72 h					
Luxor	0.35 (0.106–0.587)	1.68 (0.968–7.178)	1.884 ± 0.557	1.8	2.1
Assuit	0.18 (0.044–0.3)	0.87 (0.502–4.34)	1.857 ± 0.582	1.1	1.5
Giza	0.16 (0.037–0.251)	0.56 (0.35–2.621)	2.319 ± 0.791	1	

LC_50_ = Lethal Concentration 50%, LC_90_ = Lethal Concentration 90%, RR_50_ = Resistance Ratio 50%, RR_90_ = Resistance Ratio 90%. Significant differences were determined based on non-overlapping 95% confidence intervals (*p* < 0.05). Resistance ratios were calculated relative to the most susceptible population (Giza).

**Table 2:** Toxicity and resistance ratio of chlorfenapyr on different populations of *Tuta absoluta* second-instar larvae under laboratory conditions after 24, 48 and 72 h of exposure.

Population	LC_50_ (μg/mL) (95% CL)	LC_90_ (μg/mL) (95% CL)	Slope ± SE	RR_50_	RR_90_
After 24 h					
Luxor	126.76 (101.495–160.009)	268.12 (199.251–547.154)	3.939 ± 0.928	3.1	1.8
Assuit	98.65 (81.303–123.179)	196.77 (149.048–372.609)	4.274 ± 0.960	2.09	1.7
Giza	47.156 (28.842–60.832)	114.26 (85.019–242.35)	3.334 ± 0.902	1	
After 48 h					
Luxor	115.15 (87.456–153.125)	301.48 (206.819–772.201)	3.066 ± 0.739	3.02	1.4
Assuit	85.86 (70.648–103.643)	163.99 (129.333–268.128)	4.560 ± 0.953	1.84	1.2
Giza	46.67 (22.298–63.171)	140.87 (96.094–540.993)	2.671 ± 0.849	1	
After 72 h					
Luxor	94.19 (67.755–123.939)	263.65 (182.136–638.86)	2.867 ± 0.681	3.37	1.8
Assuit	70.95 (57.67–84.92)	134.086 (107.778–202.145)	4.636 ± 0.907	1.8	1.4
Giza	39.34 (19.479–52.396)	99.29 (73.631–219.239)	3.187 ± 0.933	1	

LC_50_ = Lethal Concentration 50%, LC_90_ = Lethal Concentration 90%, RR_50_ = Resistance Ratio 50%, RR_90_ = Resistance Ratio 90%. Significant differences were determined based on non-overlapping 95% confidence intervals (*p* < 0.05). Resistance ratios were calculated relative to the most susceptible population (Giza).

**Table 3:** Toxicity and resistance ratio of indoxacarb on different populations of *Tuta absoluta* second-instar larvae under laboratory conditions after 24, 48 and 72 h of exposure.

Population	LC_50_ (μg/mL) (95% CL)	LC_90_ (μg/mL) (95% CL)	Slope ± SE	RR_50_	RR_90_
After 24 h					
Luxor	139.50 (127.644–156.593)	187.77 (164.521–260.953)	2.6 ± 0.92	3.2	1.8
Assuit	95.53 (76.557–124.846)	227.99 (161.816–485.881)	1.892 ± 0.723	2.20	2.2
Giza	43.36 (28.876–55.224)	104.01 (76.388–242.207)	1.373 ± 0.921	1	
After 48 h					
Luxor	116.08 (98.14–130.367)	175.50 (150.168–278.481)	1.139 ± 2.001	3.04	2.3
Assuit	83.34 (66.318–106.639)	200.26 (144.637–400.68)	1.766 ± 0.700	2.2	2.6
Giza	38.07 (26.278–47.203)	77.77 (61.162–131.777)	1.132 ± 1.026	1	
After 72 h					
Luxor	107.03 (86.141–119.825)	160.47 (139.263–243.247)	1.285 ± 2.097	3.2	2.5
Assuit	68.64 (54.743–84.778)	151.58 (115.163–263.275)	1.725 ± 0.750	2.04	2.3
Giza	33.66 (22.662–41.714)	64.59 (51.596–103.08)	1.527 ± 1.144	1	

LC_50_ = Lethal Concentration 50%, LC_90_ = Lethal Concentration 90%, RR_50_ = Resistance Ratio 50%, RR_90_ = Resistance Ratio 90%. Significant differences were determined based on non-overlapping 95% confidence intervals (*p* < 0.05). Resistance ratios were calculated relative to the most susceptible population (Giza).

**Table 4:** Toxicity and resistance ratio of emamectin benzoate on different populations of *Tuta absoluta* second-instar larvae under laboratory conditions after 24, 48 and 72 h of exposure.

Population	LC_50_ (μg/mL) (95% CL)	LC_90_ (μg/mL) (95% CL)	Slope ± SE	RR_50_	RR_90_
After 24 h					
Luxor	0.56 (0.325–0.908)	2.73 (1.487–11.059)	1.854 ± 0.431	2.1	1.9
Assuit	0.33 (0.124–0.587)	2.51 (1.223–19.408)	1.464 ± 0.411	1.2	1.7
Giza	0.27 (0.106–0.442)	1.43 (0.803–6.114)	1.761 ± 0.475	1	
After 48 h					
Luxor	0.37 (0.19–0.593)	1.83 (1.019–7.461)	1.846 ± 0.462	1.8	1.8
Assuit	0.22 (0.055–0.407)	1.69 (0.857–12.54)	1.463 ± 0.440	1.1	1.7
Giza	0.21 (0.066–0.341)	1.0 (0.58–4.08)	1.863 ± 0.537	1	
After 72 h					
Luxor	0.26 (0.105–0.413)	1.24 (0.71–5.192)	1.865 ± 0.511	1.4	1.7
Assuit	0.18 (0.05–0.293)	0.77 (0.462–3.111)	2.000 ± 0.610	1	1.03
Giza	0.18 (0.05–0.289)	0.75 (0.447–3.331)	2.053 ± 0.646	1	

LC_50_ = Lethal Concentration 50%, LC_90_ = Lethal Concentration 90%, RR_50_ = Resistance Ratio 50%, RR_90_ = Resistance Ratio 90%. Significant differences were determined based on non-overlapping 95% confidence intervals (*p* < 0.05). Resistance ratios were calculated relative to the most susceptible population (Giza).

**Table 5:** Toxicity and resistance ratio of spinetoram on different populations of *Tuta absoluta* second-instar larvae under laboratory conditions after 24, 48 and 72 h of exposure.

Population	LC_50_ (μg/mL) (95% CL)	LC_90_ (μg/mL) (95% CL)	Slope ± SE	RR_50_	RR_90_
After 24 h					
Luxor	1.87 (0.33–3.53)	11.48 (5.15–3988.99)	1.625 ± 0.658	1.61	2.22
Assuit	1.19 (0.042–2.15)	6.75 (3.44–1070.38)	1.697 ± 0.710	1.02	1.3
Giza	1.16 (0.12–1.98)	5.18 (2.91–101.81)	1.973 ± 0.766	1	
After 48 h					
Luxor	1.33 (0.15–2.31)	6.75 (3.55–285.01)	1.813 ± 0.712	1.53	2.11
Assuit	0.921 (0.04–1.58)	3.52 (2.09–59.31)	2.201 ± 0.906	1.1	1.1
Giza	0.869 (0.02–1.491)	3.18 (1.90–60.27)	2.273 ± 0.960	1	
After 72 h					
Luxor	0.94 (0.06–1.54)	3.2 (1.96–36.30)	2.402 ± 0.972	1.5	1.5
Assuit	0.68 (0–1.31)	2.84 (1.55–3297.61)	2.056 ± 0.983	1.1	1.1
Giza	0.63 (0–1.21)	2.35 (1.22–3,318,312,584)	2.273 ± 0.960	1	

LC_50_ = Lethal Concentration 50%, LC_90_ = Lethal Concentration 90%, RR_50_ = Resistance Ratio 50%, RR_90_ = Resistance Ratio 90%. Significant differences were determined based on non-overlapping 95% confidence intervals (*p* < 0.05). Resistance ratios were calculated relative to the most susceptible population (Giza).

**Table 6:** Activity of α-esterases (EST–α–NA), Cytochrome P450 (PCMAN–demethylase monooxygenase), and GST (CDNB) enzymes in different populations of *Tuta absoluta*.

Populations	EST–α–NA ^1^	Relative Increase	Cytochrome P450 ^2^	Relative Increase	GST–CDNB ^3^	Relative Increase
Luxor	28.78 ± 6.87 A	1.4	198.98 ± 7.78 a	6.5	392.42 ± 11.4 A	1.5
Assuit	24.962 ± 1.81 A	1.2	75.54 ± 10.8 b	2.5	307.24 ± 14.9 B	1.2
Giza	20.53 ± 1.55 A	1.0	30.74 ± 7.02 c	1.0	262.16 ± 9.1 C	1.0
*p* value	0.1314		<0.001		<0.001	

^1^ Enzyme activity (nmol/mg protein/min) ± SD. ^2^ (PCMAN–demethylase monooxygenase) activity (nmol/mg protein/min) ± SD. ^3^ Enzyme activity (nmol/mg protein/min) ± SD. a–c and A–C: The difference among uppercase and lowercase return to find the differences/significancy between the activity of enzymes in the three governorates.

**Table 7:** Analysis of variance for enzyme activities as affected by location, ANOVA.

S.O.V.	*d.f.*	EST–α–NA			Cytochrome P450			GST–CDNB		
		MS	F Value	*p* Value	MS	F Value	*p* Value	MS	F Value	*p* Value
Location	2	51.141 NS	2.90	0.131	22,774.586 **	301.71	<0.001	13,127.753 **	90.58	<0.001
Error	6	17.625		-	75.483		-	144.927		-

NS = not significant (*p* > 0.05); ** = highly significant (*p* < 0.01).

## Data Availability

The original contributions presented in this study are included in the article. Further inquiries can be directed to the corresponding author.
